# Burkholderia pseudomallei Δ*tonB* Δ*hcp1* Live Attenuated Vaccine Strain Elicits Full Protective Immunity against Aerosolized Melioidosis Infection

**DOI:** 10.1128/mSphere.00570-18

**Published:** 2019-01-02

**Authors:** Nittaya Khakhum, Preeti Bharaj, Julia N. Myers, Daniel Tapia, Paul B. Kilgore, Brittany N. Ross, David H. Walker, Janice J. Endsley, Alfredo G. Torres

**Affiliations:** a Department of Microbiology and Immunology, University of Texas Medical Branch, Galveston, Texas, USA; b Department of Pathology, University of Texas Medical Branch, Galveston, Texas, USA; University of Maryland School of Medicine

**Keywords:** *Burkholderia pseudomallei*, melioidosis, live attenuated vaccine, *tonB*, *hcp1*, Th17, Th1, humoral immunity

## Abstract

Burkholderia pseudomallei is a Gram-negative facultative intracellular bacterium and the causative agent of melioidosis, a severe infectious disease found throughout the tropics. This organism is closely related to Burkholderia mallei, the etiological agent of glanders disease which primarily affects equines. These two pathogenic bacteria are classified as Tier 1 select agents due to their amenability to aerosolization, limited treatment options, and lack of an effective vaccine. We have previously successfully demonstrated the immunogenicity and protective efficacy of a live attenuated vaccine strain, B. mallei *ΔtonB Δhcp1* (CLH001). Thus, we applied this successful approach to the development of a similar vaccine against melioidosis by constructing the B. pseudomallei *ΔtonB Δhcp1* (PBK001) strain. C57BL/6 mice were vaccinated intranasally with the live attenuated PBK001 strain and then challenged with wild-type B. pseudomallei K96243 by the aerosol route. Immunization with strain PBK001 resulted in full protection (100% survival) against acute aerosolized melioidosis with very low bacterial burden as observed in the lungs, livers, and spleens of immunized mice. PBK001 vaccination induced strong production of B. pseudomallei-specific serum IgG antibodies and both Th1 and Th17 CD4^+^ T cell responses. Further, humoral immunity appeared to be essential for vaccine-induced protection, whereas CD4^+^ and CD8^+^ T cells played a less direct immune role. Overall, PBK001 was shown to be an effective attenuated vaccine strain that activates a robust immune response and offers full protection against aerosol infection with B. pseudomallei.

**IMPORTANCE** In recent years, an increasing number of melioidosis cases have been reported in several regions where melioidosis is endemic and in areas where melioidosis had not commonly been diagnosed. Currently, the estimated burden of disease is around 165,000 new cases annually, including 89,000 cases that have fatal outcomes. This life-threatening infectious disease is caused by B. pseudomallei, which is classified as a Tier 1 select agent. Due to the high case fatality rate, intrinsic resistance to multiple antibiotic treatments, susceptibility to infection via the aerosol route, and potential use as a bioweapon, we have developed an effective live attenuated PBK001 vaccine capable of protecting against aerosolized melioidosis.

## Abstract (precis)

In recent years, an increasing number of melioidosis cases have been reported in several regions where melioidosis is endemic and in areas where melioidosis had not commonly been diagnosed. Currently, the estimated burden of disease is around 165,000 new cases annually, including 89,000 cases that have fatal outcomes. This life-threatening infectious disease is caused by B. pseudomallei, which is classified as a Tier 1 select agent. Due to the high case fatality rate, intrinsic resistance to multiple antibiotic treatments, susceptibility to infection via the aerosol route, and potential use as a bioweapon, we have developed an effective live attenuated PBK001 vaccine capable of protecting against aerosolized melioidosis.

## INTRODUCTION

The facultative intracellular Gram-negative bacterium Burkholderia pseudomallei is the etiologic agent of melioidosis, a severe disease associated with a high mortality rate in the tropics (1, 2). B.pseudomallei is present in soil and water of environmentally suitable areas, including regions in South East Asia and Northern Australia where the organism is endemic. These areas are also associated with the highest number of reported cases and deaths due to melioidosis disease (2). Humans and animals can be infected through skin abrasions, inhalation, or ingestion (3). The spectrum of disease caused by B. pseudomallei infection ranges from acute to chronic infections, and relapse is common (5 to 28%) and occurs after long-term antibiotic treatment (1). The high mortality rate (10 to 50% worldwide and 35% in Thailand) of this disease is often caused by delays in proper treatment or due to difficulties in clinical recognition and laboratory diagnosis (3–5). Because of the high mortality rate, intrinsic resistance to multiple antibiotic treatments, low infectious dose, susceptibility to infections via the aerosol route, and its biothreats potential, it is crucial to develop an effective vaccine capable of protecting against natural or intentional infections.

Live attenuated vaccines have been shown to be the most effective way of providing complete protection and long-lasting humoral and cell-mediated immune responses, especially against intracellular pathogens (6–8). Most of the current B. pseudomallei live attenuated vaccine candidates have demonstrated partial protection (9–14). Complete protection has been demonstrated in a small subset of those live attenuated strains tested in murine models of infection, but most candidates lack defined cellular or humoral immune responses, and the persistence of such vaccines is still a concern (7, 15). Previous reports of complete protection against B. pseudomallei suggest that engagement of both innate and adaptive immune responses is required to achieve sterilizing immunity, especially in organs targeted by this pathogen.

The ability of pathogenic bacteria to acquire metals, including iron, is essential for survival, particularly in an intracellular environment. Bacteria require iron as a growth factor to perform metabolic functions, and sequestration of iron by the host serves as an innate immune defense mechanism to limit access to this metal by pathogens. This can be overcome by bacteria secreting siderophores that have higher affinities for iron than host iron binding proteins and molecules (16). The uptake of ferric siderophore complexes by *Burkholderia* species require different outer membrane receptors that interact with an inner membrane TonB complex (TonB, ExbB, and ExbD) (17, 18). Previously, our group demonstrated that disruption of *tonB* reduces the virulence of Burkholderia mallei and Burkholderia cenocepacia both *in vitro* and *in vivo* (19, 20).

Hemolysin-coregulated protein 1 (Hcp1) is a membrane-associated protein and a component of the type VI secretory system (T6SS) apparatus (21). The T6SS was shown to play an important role in the intracellular lifestyle of *Burkholderia* by inducing cell fusion, macrophage cytotoxicity, and induction of immunosuppressive cytokines IL-10 and TGF-β (21–24). Hcp1 was previously demonstrated to form the hexameric rings that assemble into a needle-like structure, important for the secretion of T6S effectors during infection (25).

Our laboratory previously constructed the B. mallei Δ*tonB* Δ*hcp1* double mutant (CLH001) by deleting the iron transport energizer, the *tonB* gene, as well as the T6SS component, the *hcp1* gene. This mutant is deficient in the ability to acquire iron and its capacity for cell-to-cell spread, leading to attenuation while maintaining its protective immunity capacity (26). Following this successful approach, our goal was to create a species-specific vaccine candidate against B. pseudomallei. In this study, we constructed a B. pseudomallei Δ*tonB* Δ*hcp1* (PBK001) vaccine strain, examined immune responses, and evaluated protective ability using an inhalational mouse model of melioidosis infection.

## RESULTS

### Evaluation of the protective capacity of B. pseudomallei PBK001 against aerosol infection.

**FIG 1:**
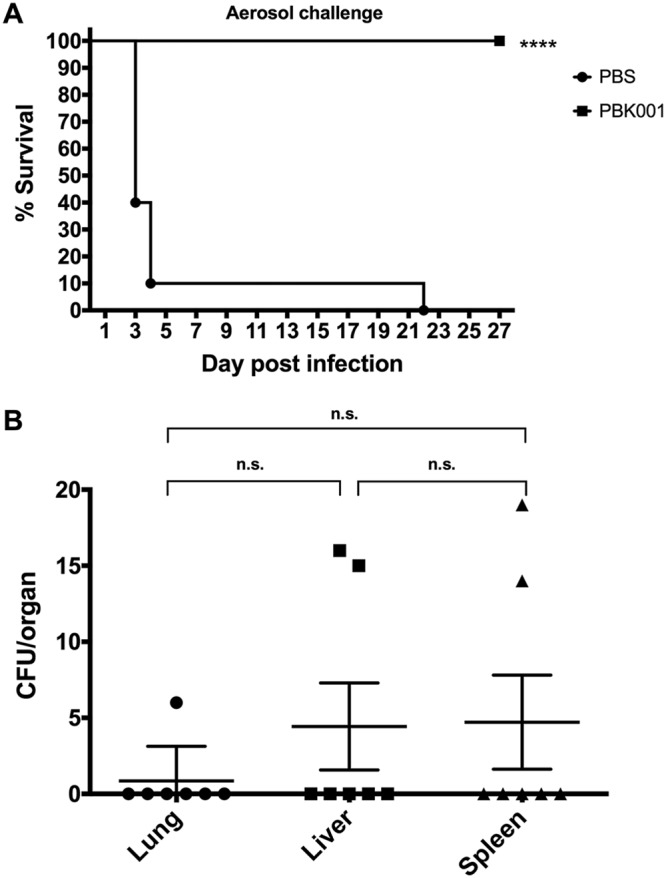
Prime and boosts with PBK001 vaccine provided 100% protection with low bacterial burden in organs. (A) C57BL/6 mice were i.n. immunized with either PBS (*n* = 10) or strain PBK001 at 1.5 × 10^4^ CFU (*n* = 10). Three weeks after the last boost, the mice were challenged with B. pseudomallei K96243 strain via the aerosol route. Survival was analyzed using a log rank (Kaplan Meier) test (****, *P* < 0.0001). (B) Bacterial burden in lungs, livers, and spleens (*n* = 7) of surviving mice at day 27 postchallenge. n.s., not significant.

C57BL/6 mice were intranasally vaccinated with a three-dose regimen of PBS or strain PBK001 at 2-week intervals and then challenged with 6.9 to 11.6 LD_50_ of B. pseudomallei K96243, 21 days after the last immunization. Following challenge with B. pseudomallei K96243, mice were monitored for survival and changes in body weight for 27 days postinfection (dpi). At the end of the study, mice vaccinated with strain PBK001 exhibited 100% survival, whereas nine of ten PBS-vaccinated mice succumbed to infection by day 4 and the tenth mouse died on day 21, as shown in Fig. 1A. On day 27 dpi, the lungs, livers, and spleens of surviving mice (*n* = 10) were harvested and processed for CFU enumeration (*n* = 7) and histopathology evaluation (*n* = 3). No bacteria were detected in all three organs evaluated from five animals; however, some bacteria (limit of detection, <10 CFU/organ) were detected in the organs from two of the PBK001-vaccinated mice (Fig. 1B**)**. The survival study and bacterial load determinations indicated that immunization with strain PBK001 protected mice from aerosolized melioidosis and significantly reduced the level of B. pseudomallei infection in the three organs analyzed.

### Humoral immune response following B. pseudomallei PBK001 immunization.

**FIG 2:**
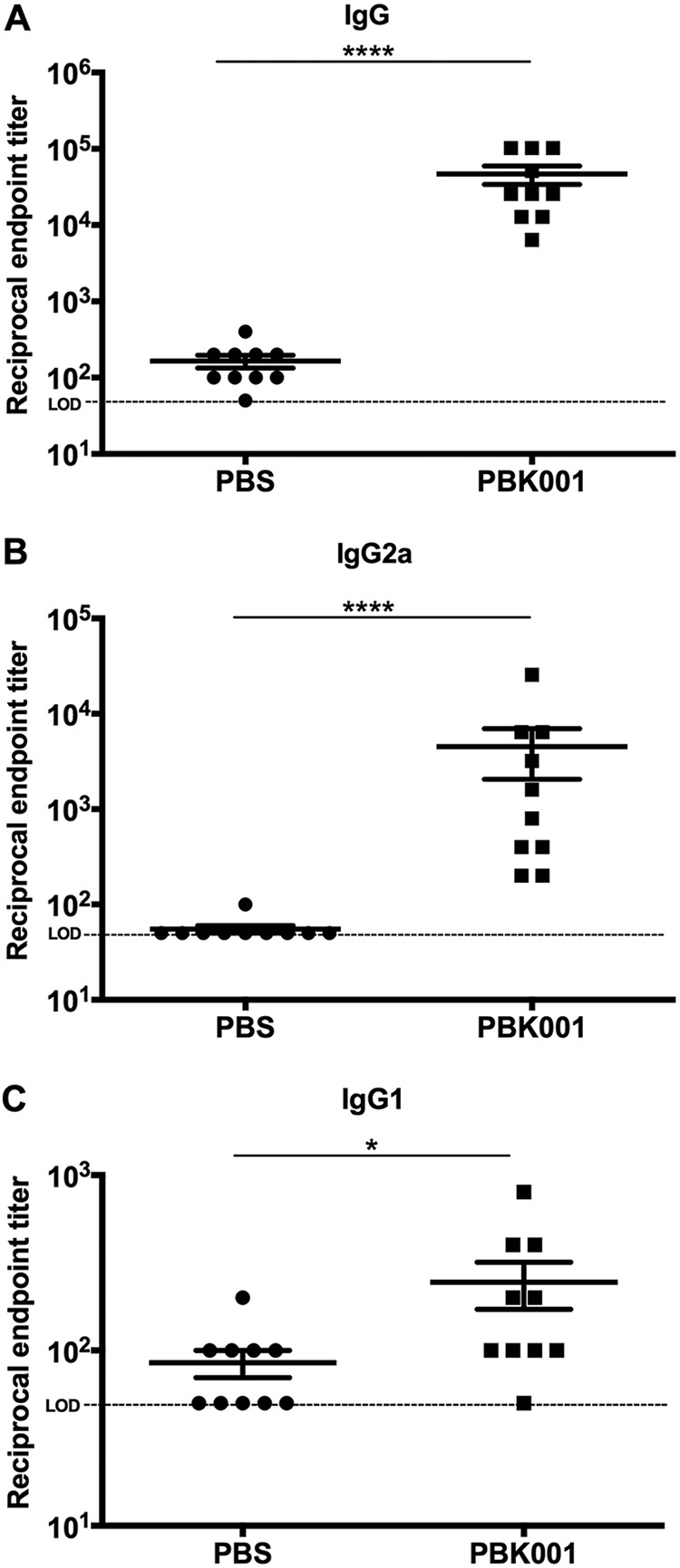
Detection of B. pseudomallei-specific antibody titers. C57BL/6 mice (*n* = 10 per group) were immunized with either PBS (control) or PBK001 vaccine (1.5 × 10^4^ CFU). Serum samples were collected from individual vaccinated mice 2 weeks after the last boost, and serum IgG (A), IgG1 (B), and IgG2a (C) against B. pseudomallei whole cells were analyzed by indirect ELISAs. Antibody levels were reported as the reciprocal of the highest titer giving an optical density (OD) reading of at least the mean plus 2 SD of baseline sera and analyzed via the Mann-Whitney test. The limit of detection (LOD) is 50 (horizontal dotted line). Bars represent means with standard error of the mean (SEM). Values that are significantly different are indicated by bars and asterisks as follows: ****, *P* < 0.0001; *, *P* < 0.05.

Sera from individual PBS and PBK001-vaccinated mice were analyzed for antibody responses to irradiated B. pseudomallei K96243 whole-cell lysate (WCL). Total IgG and IgG2a and IgG1 subclasses were determined using indirect ELISA. The sera from individual mice that received a prime and two boosts of PBK001 vaccine showed significantly higher reciprocal endpoint titers (mean ± standard errors of the means [SEM]) for IgG (46,720 ± 12,730), IgG2a (4,520 ± 2,464), and IgG1 (231 ± 73) compared to PBS-vaccinated mice (Fig. 2A to C). In addition, the IgG2a/IgG1 ratio was calculated to be 19.5 for the PBK001 vaccine. PBK001 vaccination generated high levels of B. pseudomallei-specific IgG and higher levels of IgG2a, indicative of Th1-biased immune response.

### Development of cellular immune response induced by PBK001 vaccination.

**FIG 3:**
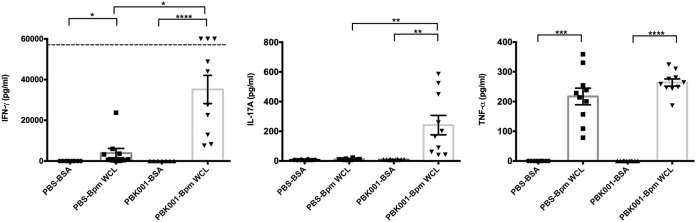
PBK001 immunization induces cellular immune response. On day 21 after the second boost, the spleens from PBS- or PBK001-vaccinated mice were harvested and restimulated with BSA or heat-killed B. pseudomallei K96243 whole-cell lysate (WCL) for 3 days. IFN-γ, IL-17A, and TNF-α production in cell supernatants was measured using ELISA. The data were analyzed using one-way ANOVA (Kruskal-Wallis test). Bars represent means with standard errors of the means (SEM). The horizontal broken line represents the limit of detection. Statistical significance: ****, *P* < 0.0001; ***, *P* < 0.001; **, *P* < 0.01; *, *P* < 0.05.

To assess the role of cellular immune responses in PBK001 vaccine-induced protection, the spleens of PBS- and PBK001-vaccinated mice were collected on day 21 after the last vaccination. Single-cell suspensions of splenocytes were seeded and restimulated with BSA or heat-killed B. pseudomallei K96243 WCL for 72 h. Splenocytes from PBK001-vaccinated mice significantly increased production of IFN-γ, TNF-α, and IL-17A after stimulation with specific antigen (B. pseudomallei WCL) compared to nonspecific stimuli (BSA) (Fig. 3). Consistent with nonspecific innate immune activation by WCL, some IFN-γ was generated from spleen cells from PBS-vaccinated mice in response to antigen. These nonspecific responses were significantly lower than the antigen-specific response observed with splenocytes from the PBK001-vaccinated mice (*P* = 0.0273). Significant activation of TNF-α by WCL was observed in splenocytes from both PBS and PBK001 groups, though further increased production was observed in the vaccinated group. The nonspecific TNF-α production is consistent with activation of splenic macrophage innate immunity via pathogen recognition receptor binding of WCL components. These results demonstrated that the PBK001 vaccine induced an antigen-specific cellular response by production of effector cytokines characteristic of Th1 (IFN-γ) and Th17 (IL-17A) immune responses.

### Role of T cell populations in PBK001 vaccine-induced protection.

**FIG 4:**
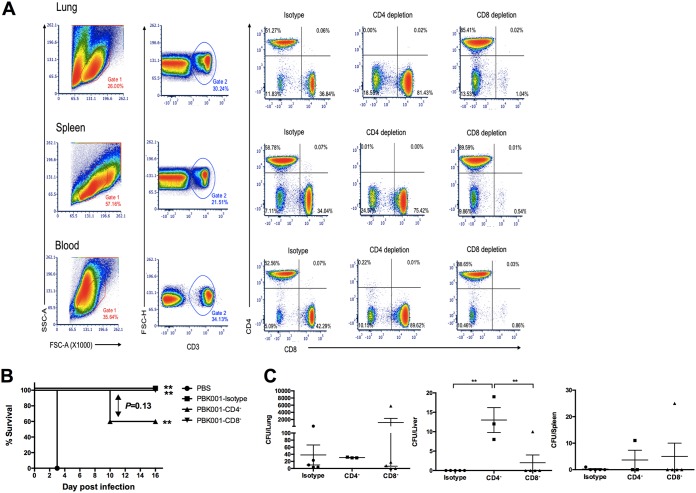
Role of T cell populations in PBK001 vaccine-induced protection. (A) Confirmation of T lymphocyte depletion using three-color FC analysis of cells from intact, CD4-depleted, and CD8-depleted mice. Cells (10^6^) from lung, spleen, or blood were stained with APC-anti-CD3, PE-anti-CD4, and PerCP Cy5.5-anti-CD8 antibodies after *in vivo* depletion. Results are shown on density plots with a logarithmic scale. The percentages of T lymphocytes from each organ of isotype and depleted sets are shown in each quadrant. (B) C57BL/6 mice (*n* = 5/group) were immunized with strain PBK001 or PBS. Two weeks after the second boost, mice were treated with rat IgG2b isotype control, anti-mouse CD4 and anti-mouse CD8α and then challenged with 3 LD_50_ of B. pseudomallei K96243. Mice were monitored daily and survival (percent) differences were determined using a log rank (Kaplan-Meier) test. (C) Bacterial burden in lung, liver, and spleen was determined on day 16. The difference of CFU/organ from each was compared using one-way ANOVA (Tukey’s test). Bars represent means with standard errors of the means (SEM). **, *P* < 0.01.

To determine the protective role of either CD4^+^ or CD8^+^ T cells in PBK001-immunized mice, vaccinated animals were depleted of individual T cell populations prior to challenge with B. pseudomallei. Two weeks after the last vaccination, mice received an i.p. injection with either isotype control or with antibodies specific to murine CD4 or CD8 T cells, 3 days before challenge (day −3) or on the day of challenge (day 0), and depletion was continued every 3 days after challenge (days +3, +6, +9, and +12). The depletion efficiencies were confirmed by flow cytometric analysis of CD4^+^ and CD8^+^ T cell populations in an age- and gender-matched group of noninfected mice. The results demonstrate that the depletion protocol resulted in 99.78 to 100% depletion of CD4^+^ and 98.9 to 99.5% of CD8^+^ T cells in the lung, spleen, and peripheral blood (Fig. 4A).

As predicted by the percent animal survival (Fig. 4B), mice that received PBS succumbed to infection at 3 days postinfection (dpi), while PBK001-immunized mice without T cell depletion (isotype control) showed 100% survival at 16 dpi. The survival of PBK001-CD4^+^ and PBK001-CD8^+^ depleted mice showed 60% and 100% survival on 16 dpi, respectively. At the end of study, the lungs, livers, and spleens of surviving mice were harvested and processed for CFU enumeration to determine the effect T cell depletion had on controlling the bacterial burden. Depletion of CD4^+^ T cells increased bacterial burden in liver compared to isotype control and CD8^+^ depleted mice (Fig. 4C), though overall liver burden was very low. These data showed a moderate contribution of CD4^+^ T cells to cell-mediated immunity against B. pseudomallei, while CD8^+^ T cells do not appear to contribute significantly to vaccine-induced protection.

### Gross pathology and histopathology analysis of infected tissues.

**FIG 5:**
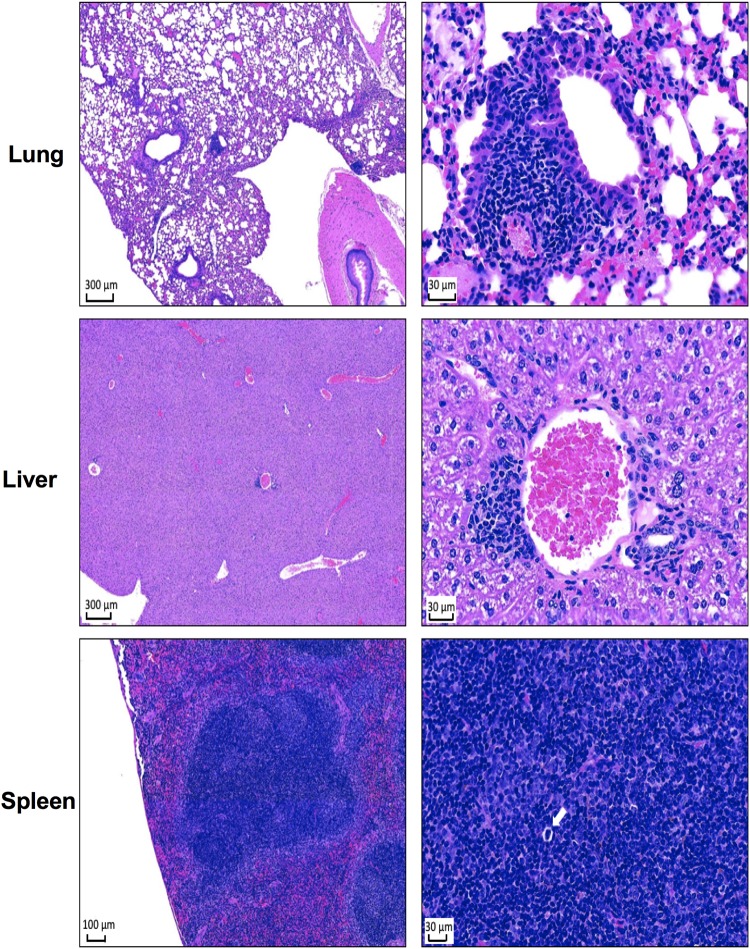
Histopathology analysis of PBK001-vaccinated mouse tissues after aerosol challenged with B. pseudomallei K96243. At the end of study, organs were harvested from three mice. The tissues were fixed and stained with H&E. Stained tissues display findings seen in lung, liver, and spleen 27 days after challenge. The left figures represent images of 4× (bar = 300 μm) (lung and liver) and 10× (bar = 100 μm) (spleen) magnification, and the right figures represent 40× (bar = 30 μm) magnification. The white arrow indicates a hematopoietic lesion.

To determine the extent of tissue damage, gross pathology and histological analysis of tissues (lung, liver, and spleen) from surviving mice (*n* = 3) exposed to aerosolized B. pseudomallei K96243 were evaluated at 27 dpi. Gross pathological observation of all the organs analyzed indicated normal size and unremarkable pathology (data not shown). For histopathology, the numbers of inflammatory foci from three mice were quantified by counting 10 fields of ×40 magnification per organ and reported as means ± standard deviations (SD). Representative images of H&E-stained lungs, livers, and spleens from PBK001-vaccinated mice are shown in Fig. 5. Lung sections showed areas of lymphocyte-rich inflammation surrounding the bronchoalveolar tree estimated as 3 ± 1.4 foci per ten ×40 magnification fields. Liver sections also showed multifocal infiltrates of mixed inflammatory cells (1 ± 0.63) (lymphocyte rich) occasionally associated with apoptotic hepatocytes. Spleen sections showed minimal to unremarkable pathological damage. Prominent periarteriolar lymphoid sheaths (PALS) and increased hematopoiesis were found in the spleens of surviving animals. Overall, the histopathologic analysis showed minimal change in the organs of surviving vaccinated mice after aerosol challenge, supporting our findings that vaccination prevents pathogen persistence and therefore causing minimal pathological changes in target organs.

## DISCUSSION

Melioidosis is a fatal disease that is increasingly recognized to be associated with high morbidity and mortality, especially in tropical regions worldwide. No licensed vaccine is available to prevent disease caused by B. pseudomallei infection (4); therefore, an effective vaccine against this pathogen is urgently needed for both public health and biodefense purposes. Live attenuated vaccine candidates against melioidosis have been developed and tested mainly in BALB/c and C57BL/6 mouse models using various targeted single gene mutants of key enzymes in biosynthetic pathways, including *aroB* (11), *aroC* (27), *purN* (12), *purM* (7, 12, 15), *serC* (28), and *ilvl* (2D2) (9, 29), or the Bsa type III secretion system gene *bipD* (10). However, most of the studies with these mutant strains were performed in the BALB/c mouse model, which is more susceptible to B. pseudomallei infection, and therefore, the results showed that most vaccines were insufficient to generate complete protection against wild-type challenge (9–12, 15, 28, 29). In contrast, C57BL/6 mice are a more suitable model for infection and more representative of chronic human melioidosis (30–32). Further, C57BL/6 mice have been more recently used in the assessment of several live attenuated and subunit vaccines, and complete protection has been demonstrated against various challenge strains (7, 14, 27). We have previously demonstrated that a B. mallei *ΔtonB Δhcp1* vaccine was effective and safe and generated full protection against glanders disease (26). Therefore, we constructed the B. pseudomallei double mutant PBK001 by targeting the same genes deleted in our B. mallei vaccine and following a similar methodology used to construct the CLH001 vaccine. The PBK001 strain displayed attenuated characteristics like CLH001, as demonstrated by the inability to colonize (data not shown) and cause infection in C57BL/6 mice and provided strong evidence that this vaccine candidate was effective in protecting mice against aerosolized B. pseudomallei infection.

Following vaccination with strain PBK001, C57BL/6 mice were challenged with aerosolized B. pseudomallei K96243. We used the nose-only aerosol system to establish an inhalational infection of B. pseudomallei that most closely recapitulates human inhalational cases. Aerosol provides the most effective means to test an inhalational infection and mimics the pathogen’s organ dissemination patterns (33, 34). Importantly, an assessment of the bacterial burden at 27 dpi demonstrated that mice receiving PBK001 vaccine had successfully cleared the infection in the lung, liver, and spleen. In addition, residual damage was minimal in these organs as analyzed by histopathologic analysis. However, the mechanism of bacterial control mediated by the vaccine in the lungs needs further investigation.

To achieve complete protection and bacterial clearance that can prevent melioidosis, it is postulated that strong humoral and cellular immune responses are required, since B. pseudomallei can survive and replicate within host cells. The role of B. pseudomallei-specific CD4^+^ T cells was shown to be important for host resistance, and IFN-γ-mediated immunity has a critical role for survival in humans and in murine models of infection (35–41). Our results demonstrated that vaccination using a prime and two boost regimens of PBK001 vaccine generated strong, protective, B. pseudomallei-specific serum IgG. Also, the increased IgG2a/IgG1 ratio of >1 indicated that the vaccine provided a strong Th1-biased immune response. Our data also showed that PBK001 vaccination induced the activation of cellular responses as demonstrated by the production of IFN-γ and IL-17A by the splenocytes of vaccinated mice upon antigen restimulation. Although the role of IL-17 is still unclear with regard to vaccine-induced protection against B. pseudomallei, the production of IFN-γ regulates intracellular killing and is associated with lower bacterial burdens in organs after *Burkholderia* infection (35, 42, 43). On the other hand, IL-17 has a critical role in vaccine-induced immunity against bacterial infections, primarily by induction of chemokines to recruit protective Th1 cells, neutrophils, macrophages, and enhanced phagocytic killing (44).

Even more interesting were the results from our T cell depletion study, which indicate that T cells do not have a primary immune role in protection from challenge. An important helper function of CD4^+^ T cells is likely required for development of the protective humoral immune response that was generated by strain PBK001. At the time of challenge, however, only a moderate cell-mediated immune role for CD4^+^ T cells was observed, while CD8^+^ T cells appeared to be dispensable. The survival and liver bacterial load results of T cell-depleted mice suggest that CD4^+^ T cells may play a more important complementary role in protection than CD8^+^ T cells. Alternatively, CD4^+^ and CD8^+^ T cell subsets may have redundant roles to mediate protection through production of effector cytokines such as IFN-γ. In addition to supporting development of strong humoral memory during immune priming, CD4^+^ T cells may also play a complementary role in immunity as the primary source of IL-17. Further, we can speculate that the remaining bacteria in tissues could be kept under control or cleared if the memory immune response is lasting. Altogether, our results suggested that the humoral immune response served as a crucial component for PBK001 vaccine-induced protection and that T cells are more likely to play a complementary role in protective immunity.

Our results are consistent with the evidence that patients who survive melioidosis have higher serum IgG titers than patients who succumbed to infection (45). Some studies have demonstrated that antibodies against polysaccharides also play a role in B. pseudomallei protection by promoting opsonization and inducing phagocytic killing of bacteria *in vitro* (46–51). Moreover, B cells are required for another function, such as amplification of the IFN-γ response by T cells via a TNF-α-mediated mechanism during infection (52). Further studies (e.g., passive transfer) are required to conclusively demonstrate the roles of antibodies, B cells, and other associated immune cells in the PBK001 protection mechanism.

Several live attenuated vaccine strains against B. pseudomallei infection have demonstrated that the distinct immune response outcomes are dependent on the lipopolysaccharide (LPS) type backbone expressed by B. pseudomallei (49, 53, 54). In our study, the humoral immune response played an important role for PBK001-induced protection, whereas T cells played a minor role, despite the reduction in survival when CD4^+^ T cells were depleted (no statistically significant difference). Our results are similar to the data seen with Bp82 vaccine immunization that induced protective immunity by generating an effective humoral immune response, which was independent of both CD4^+^ and CD8^+^ T cells (7). However, another B. pseudomallei live attenuated vaccine, 2D2, generated incomplete immunity and elicited protection mediated by CD4^+^ T cells but not CD8^+^ T cells when evaluated in BALB/c mice (29). Recent studies have also corroborated the idea that B. pseudomallei strain 576a mutant vaccine 2D2 strain, containing LPS type B, was exceptional in inducing innate and T cell responses. In contrast, LPS type A from strain 1026b (Bp82 backbone) was a weak inducer of T cell-mediated immunity (54). Our data further confirm this finding as shown with the PBK001 vaccine strain, which is constructed from the parent strain K96243, which also contains LPS type A.

### Conclusions.

This study describes a safe and attenuated B. pseudomallei *ΔtonB Δhcp1* mutant vaccine strain that generated full protection against a lethal dose of aerosolized melioidosis, producing almost complete sterilized immunity. The value of this vaccine may increase if protection against different B. pseudomallei strains and cross-protective properties against B. mallei infection can be confirmed.

## MATERIALS AND METHODS

### Ethics statements.

All animals in this study were handled in strict accordance with the recommendations in the Guide for the Care and Use of Laboratory Animals of the National Institutes of Health. Mice were housed in microisolator cages under pathogen-free conditions, provided with rodent feed and water *ad libitum*, and maintained on a 12-h light cycle in an animal biosafety level 3 (ABSL3) laboratory, and all experimental protocols were reviewed and approved by the Institutional Animal Care and Use Committee of the University of Texas Medical Branch (protocol 0503014D) and the Animal Care and Use Review Office of the Department of the Army.

### Bacterial strains and growth conditions.

The bacterial strains used in this study are listed in Table 1. Escherichia coli was grown on Luria-Bertani (LB) agar or in LB broth containing 50 μg/ml of kanamycin. B. pseudomallei K96243 and B. pseudomallei K96243 Δ*hcp1* (CLH010) strains were grown on LB with 4% glycerol (LBG) agar for 48 h and shaken in LBG broth for 12 h. B. pseudomallei Δ*tonB* Δ*hcp1* mutant was grown in low-salt (0.5% NaCl) LB agar supplemented with 200 μM FeSO_4_·7H_2_O or low-salt LB broth for 16 h. Bacteria on agar plates were incubated at 37°C. Bacterial stocks were maintained in 20% glycerol and stored in −80°C freezer. All manipulations of B. pseudomallei strains were conducted in a CDC-approved and registered biosafety level 3 (BSL3) or CDC/USDA-approved and registered animal biosafety level 3 (ABSL3) laboratory facility at the University of Texas Medical Branch, and all experiments were performed in guidance with standard select agent operating practices.

**TABLE 1:** Bacterial strains and plasmid used in this study

Bacterial strain	Relevant features^*a*^	Reference or source
B. pseudomallei K96243	Human clinical isolate from Thailand; Ipm^s^ Caz^s^ Chl^s^ Cip^s^, Augmentin^s^ Min^r^ Gen^r^ Tmp-Smx^r^ Str^r^	56
B. pseudomallei PBK001	B. pseudomallei CLH010 with an unmarked intragenic deletion in BPSS0368 (Δ*tonB*)	This study
B. pseudomallei CLH010	B. pseudomallei K96243 with an unmarked intragenic deletion in BPSS1498 (Δ*hcp1*)	This study
E. coli S17-1 λ*pir* (pMo130-ΔBPSS1498)	Donor strain containing pMo130-Δbpss1498 plasmid; Sm^r^ Tp^r^ Pb^s^ Km^r^	23

a Ipm, imipenem; Caz, ceftazidime; Chl, chloramphenicol; Cip, ciprofloxacin; Augmentin, amoxicillin/clavulanate potassium; Min, minocycline; Gen, gentamicin; Tmp-Smx, trimethoprim-sulfamethoxazole; Str, streptomycin; Km, kanamycin; Pb, polymyxin B; Sm, streptomycin; Tp, trimethoprim.

### Construction of B. pseudomallei Δ*tonB* Δ*hcp1* mutant.

We employed a similar methodology used to construct the B. mallei *ΔtonB Δhcp1* (CLH001) live attenuated vaccine to create the B. pseudomallei *ΔtonB Δhcp1* (PBK001) vaccine strain. The PBK001 strain was constructed using a select agent compliant suicide vector allelic exchange system described previously (55). First, the B. pseudomallei K96243 Δ*hcp1* (CLH010) strain was obtained using donor strain E. coli S17-1 λ*pir* containing the plasmid pMo130-ΔBPSS1498 (provided by Mary Burtnick, University of Nevada at Reno). This plasmid created an unmarked in-frame deletion of *hcp1* (23). Five hundred microliters of E. coli donor strain were mixed with 500 μl of B. pseudomallei K96243, centrifuged, and resuspended in 100 μl of 10 mM MgSO_4_ as a mix or individually for controls. Each conjugation was spotted (25 μl) on LBG agar and incubated for 8 to 16 h at 37°C. Selection of merodiploids was performed by resuspending the mixed reactions in Dulbecco’s phosphate-buffered saline (DPBS), and the diluted solutions were plated on LBG agar containing 250 μg/ml of kanamycin to select for integration and 30 μg/ml of polymyxin B to select against the donor E. coli strain. The plates were incubated for 48 to 96 h at 37°C. Merodiploids were screened by exposing the plates to 0.45 M pyrocatechol spray solution and selecting for yellow colonies. Counterselection was performed by subculturing merodiploids in 2× yeast extract-tryptone (2×YT) broth without salt for 4 h and then plating on 2×YT agar without salt and supplemented with 15% sucrose for 48 to 72 h. The resulting colonies were then screened with pyrocatechol, and nonyellow colonies were screened by PCR to confirm deletion of *hcp1*. Mutants positive by PCR were then sequenced to confirm the mutation. Strain PBK001 was constructed by using strain CLH010 (Δ*hcp1*) and introducing the *tonB* mutation using pMo130Δ*tonB* as described previously (19). Mutant construction was performed as described above except that LBG agar was supplemented with 200 μM FeSO_4_. The Δ*hcp1* mutant was confirmed using primers as previously described (26), and the Δ*tonB* mutation was then confirmed by PCR, followed by sequencing using the following primers: forward primer, GAA TTG CTG ATC GGA TTC GT; reverse primer, TCC GTA GCT TTG CAT TTC CT.

### Immunization and aerosol challenge.

Female 6- to 8-week-old C57BL/6 mice were purchased from Charles River Laboratories (Wilmington, MA, USA) and acclimated in the ABLS3 laboratory for 5 days before vaccination. Anesthetized mice (*n* = 20) were administered with three intranasal (i.n.) vaccination regimens of 50 μl PBS or 1.5 × 10^4^ CFU of strain PBK001 at 2-week intervals (days −21, −35, and −49 prechallenge). Three weeks after the second boost (day 0), mice were challenged via aerosol with B. pseudomallei K96243 using a nebulizer concentration of ∼4.45 × 10^7^ CFU/ml. Briefly, the UTMB aerobiology facility utilized a Biaera AeroMP aerosol management platform housed within the IsoGARD class III glovebox, a nebulizer, a stainless steel dilution/delivery line, a rodent exposure chamber, a relative humidity/temperature transducer, and an impinger. Mice were placed into nose-only restraint tubes, transferred to stainless steel boxes, and loaded into rodent exposure chamber. Two groups of mice (*n* = 20) were exposed to bacteria via a three-jet nozzle collision nebulizer for 15 min. Nebulizers containing appropriate concentrations of B. pseudomallei K96243 in 10 ml of LB and samples collected from a SKC Biosampler containing 20 ml of LBG were diluted and plated to determine the presented dose (Dp). Mice received Dp of 1,070 and 1,780 CFU of B. pseudomallei K96243 from runs 1 and 2 (10 mice per run). Animal weight and survival were monitored for 27 days after aerosol challenge.

### Detection of B. pseudomallei antigen-specific antibodies.

Serum samples were collected from individual mice before and 2 weeks after the last vaccination. Briefly, whole blood was collected via retro-orbital bleeding. The blood was stored in Microvette tubes without an anticoagulant and incubated at room temperature for 30 min to permit clotting before centrifugation. Mouse serum samples were irradiated, and sterility was verified by plating on LBG with FeSO_4_. Irradiated serum from PBS- or PBK001-vaccinated C56BL/6 mice were used to determine B. pseudomallei-specific IgG and subclasses (IgG2a and IgG1) endpoint titers by indirect ELISA. The 96-well high binding microplates were coated with 10 μg/ml of irradiated B. pseudomallei K96243 in DPBS at 4°C overnight. The wells were washed twice with washing buffer (0.05% Tween 20 in 1× DPBS) and incubated with blocking buffer (0.1% Tween 20, 1% BSA, 1× DPBS) for 2 h at room temperature (RT). After blocking, plates were washed twice. Twofold dilutions of sera were made with sample diluent, added to triplicate wells, and then incubated at RT for 2 h. After the microplates were washed three times, goat anti-mouse IgG, IgG2a, or IgG1 antibody (Southern Biotech) (diluted 1:5,000) was added to each well and then incubated for 2 h. The plates were washed four times before the addition of tetramethylbenzidine (TMB) substrate solution (Invitrogen). After 15 min, 100 μl of stop solution (2 N H_2_SO_4_) was added, and the wells were read at 450 and 570 nm using a microplate reader (Biotek). The results were reported as the reciprocal of the highest titer giving an optical density (OD) reading of at least mean plus 2 SD of baseline sera. All assays were performed in triplicate, and results were shown as the mean reciprocal endpoint titer.

### *Ex vivo* stimulation assay for T cell immunity.

Spleens were collected from vaccinated mice 21 days after the last vaccination, and single-cell suspensions were isolated by passage through a 100-μm nylon cell strainer (BD Falcon) and then treated with 1× red blood cell (RBC) lysis buffer (Invitrogen). Splenocytes were seeded in 24-well plates at a concentration of 1.5 × 10^6^ cells/ml and restimulated with 2 mg/ml BSA (negative control), heat-killed B. pseudomallei K96243 WCL (1 × 10^7^ CFU/ml), or Dynabead mouse T-activator CD3/CD28 (positive control) (BD Bioscience) at 37°C and 5% CO_2_ for 72 h. Cell culture supernatants were collected and analyzed for production of secreted IFN-γ, IL-17A, and TNF-α production using ELISA kits according to the manufacturer’s instruction (Invitrogen).

### *In vivo* depletion of CD4^+^ and CD8^+^ T cells after immunization with PBK001.

Female, 6- to 8-week-old C57BL/6 mice (*n* = 20; 5 mice/group) were vaccinated using a prime and two-boost regimen with strain PBK001 or PBS as described above. The depletion was performed 2 weeks after the last vaccination using rat IgG2b isotype control (LTF-2) (catalog no. BE0090), rat anti-mouse CD4 (GK1.5) (catalog no. BE0003-1), or rat anti-mouse CD8 (YTS 169.4) (catalog no. BE0117) purchased from BioXcell. Mice were administered with 500 μg of MAb intraperitoneally (i.p.) 3 days before infection and 250 μg on the day of infection. Depletion was maintained by further administration of 250 μg of MAb every 3 days postinfection. The mice were monitored for 16 days, and then their organs were collected and plated to evaluate bacterial burden. The peripheral blood (retro-orbital), lungs, and spleens were collected from a separate but matched group of mice (2 mice/group) to allow assessment of the protocol over the period of the depletion. The efficiency of depletion was confirmed by flow cytometry analysis 24 h after staining. Animals were monitored, weighed, and recorded until the end of experiment. Flow cytometry analysis was performed on 0.1-ml portions of blood samples transferred to Microvettes coated with lithium heparin. Peripheral blood cells or single-cell tissue suspensions were incubated with Fc block (catalog no. 553142; BD Bioscience) for 5 min to block non-antigen-specific binding of immunoglobulins. APC-conjugated anti-mouse CD3 (17A2), PE-conjugated anti-mouse CD4, and PerCP-Cyanine 5.5-conjugated anti-mouse CD8α purchased from eBioscience were used for surface marker analysis of CD3, CD4, and CD8 T cells, respectively. RBCs were lysed using RBC lysis buffer. Surface-stained samples were fixed with 2% ultrapure formaldehyde diluted in PBS for 48 h before flow cytometry analysis using a BD Fortessa LSR II flow cytometer, and results were analyzed using FCS Express 6 (Glendale, CA, USA).

### Bacterial burden and histological analysis.

The lungs, livers, and spleens from surviving mice were harvested for CFU enumeration (*n* = 7) and histopathology analysis (*n* = 3). Organs from seven mice were homogenized in PBS, serially diluted, and plated on LBG agar. The colonies were counted after 48 h of incubation at 37°C. Organs from three mice were collected, fixed in 10% formalin, embedded in paraffin, and then cross-sectioned before hematoxylin and eosin (H&E) staining. The stained tissue sections were examined in a blinded fashion by a pathologist.

### Statistical analyses.

Analyses were performed using GraphPad Prism7 software (La Jolla, CA). Survival differences were compared using Kaplan-Meier survival curves, followed by a log rank test. A nonparametric *t* test (Mann-Whitney test) was used to analyze the significant difference between two groups, while one-way analysis of variance (ANOVA) was used for multiple group comparison, followed by Dunn’s multiple means or Turkey’s comparison test.
